# Language and Communication Barriers in Emergency Departments in Makkah: Physicians’ Perspective

**DOI:** 10.7759/cureus.58987

**Published:** 2024-04-25

**Authors:** Noura M Bakhsh, Omniyh A Fatani, Rawya Zeed Melybari, Raghd Alabdullah, Reem F Bahakeem, Salman H Alsharif, Jehad K Alharbi, Osamah A Fatani

**Affiliations:** 1 Emergency Medicine, King Fahad General Hospital, Makkah, SAU; 2 Medicine, Umm Al-Qura University, Makkah, SAU; 3 Medicine and Surgery, Umm Al-Qura University, Makkah, SAU; 4 Urgent Care, Ibn Sena Hospital, Makkah, SAU; 5 General Medicine, Umm Al-Qura University, Makkah, SAU

**Keywords:** emergency department, makkah, barriers, language, communication

## Abstract

Background

Communication is essential in the medical sector, particularly in the emergency departments (ED), to provide appropriate patient care. Lack of patient history and large patient numbers, cultural variations, inadequate health literacy, and language difficulties can impact effective communication.

Aim

This study aims to examine language and communication barriers experienced by ED physicians in Makkah, Saudi Arabia, as well as to determine the effect of language barriers on patient care and explore possible methods to deal with language and communication barriers.

Methods

This cross-sectional study was conducted from April 8 to June 6, 2023. A total of 136 responses were collected from ED physicians at the six Ministry of Health Hospitals (MOH) in Makkah through a validated online survey. The data analysis was implemented using RStudio (R version 4.1.1).

Result

In this study, 136 participants' data were examined. Of note, one-quarter of ED physicians (25%; n=34) under study stated that they always experienced language barriers, whereas 64.7% (n=88) of them sometimes experienced these difficulties. More than half of the ED physicians (54.4%; n=74) stated that their patients had poor outcomes because of poor communication. Among those who responded positively to poor outcomes, ED physicians’ suggestions to improve communication with patients included providing labels of the common scientific terminologies in different languages (59.6%; n=81) and providing courses to communicate in foreign languages (48.5%; n=66).

Conclusion

Exposure to language barriers among ED physicians in Makkah was slightly high. This may impact the patient’s outcomes. Therefore, strategies to improve patient-physician communication are needed.

## Introduction

Emergency Department (ED) physicians deal with difficult conditions involving death, accidents, rape, and violence [1]. Poor communication can result in unintended negative impacts on patients' health and compliance with ED physician's instructions [1].

Communication is essential in the medical sector, particularly in the ED, to offer patients high-quality treatment by comprehending their complaints and doing the necessary investigations and management [2]. It assists in the administration of effective patient care, particularly in the setting of high acuity, limited availability of patient history, and large patient numbers [3]. In addition, many health providers consider communication to be more effective when it responds to patient requirements, beliefs, and preferences. Multiple factors can impact effective communication, for example, cultural variations, inadequate health literacy, and linguistic difficulties [4]. Language barriers have been highlighted as the most significant challenge to providing adequate, appropriate, effective, and timely care to people who do not understand the local language [5].

Failure to appropriately explain the diagnosis to the patient has a significant risk that might have negative consequences. For example, patients may refuse potentially life-saving therapy or fail to comply with instructions [6]. Prior research has demonstrated that poor communication within the ED can negatively affect both the patient's experience and the safety and quality of care received [5]. Previous studies revealed that individuals with poor language competence are observed to have poor healthcare access and reflect a lower quality of care when compared to individuals competent in the main country language [7,8]. People travel to Saudi Arabia from more than 180 nations during Hajj and Umrah in Makkah; it is the site of the world's largest mass gathering [9]. Due to the presence of patients from all over the world during the Hajj and Umrah seasons in Makkah, language barriers in healthcare settings are very common. Based on a prior study done in Saudi Arabia, the most prevalent barrier experienced by medical residents was the language barrier. According to the study, managing an event with millions of people requires careful planning by ministries to ensure adequate transportation, lodging, and healthcare services [10]. There is a lack of studies regarding language barriers in ED in Saudi Arabia; therefore, this study aims to examine the language and communication barriers experienced by ED physicians in Makkah.

## Materials and methods

Study design

This was a cross-sectional survey study that was conducted from April 8, 2023, to June 6, 2023. A self-administered structured survey was distributed among ED physicians in the Ministry of Health Hospitals (MOH) in Makkah, Saudi Arabia. The study used an online questionnaire that was distributed in Google Forms in English to the targeted population (ED physicians) via social media apps (WhatsApp and Telegram) using self-administered questionnaires consisting of closed-ended questions.

Study population 

This study included ED physicians of all levels starting from residents up to consultants working in six MoH hospitals who agreed to participate in the study, while other specialties such as pediatric, obstetric, and gynecology physicians ..etc were excluded.

Data collection

Data were collected from ED physicians through an online questionnaire that consisted of closed-ended questions. No personal data or private information was collected, and all responses were kept confidential. Based on the number of physicians in the ED in Makkah hospitals, which represented 202 ED physicians, a sample size of 136 individuals was calculated by using the Raosoft sample size calculator as enough to produce a 95% confidence interval with a 5% accepted margin of error.

Questionnaire structures

The questionnaire items were developed to assess physicians' language and communication barriers in the ED. The survey items were adapted from previous studies [11-13]. A pilot study was done that included 20 participants, and the results were used to assess reliability and validity. The analysis of the reliability of internal consistency revealed a Cronbach's alpha value of 0.79.

Questionnaire validation

A panel of three experts evaluated the questionnaire's validity. To determine whether the original items were appropriate for examination, the experts modified them. The objective assigned to the subject matter experts was to evaluate each item's applicability and relevance on a four-point scale (Table 1).

**Table 1:** Validation score

Item	Score
Adequate (simple, relevant, and clear)	4
Adequate but needs minor revision	3
Needs major modification	2
Not so adequate (can be omitted)	1

The content validity index (CVI) is the proportion of all items with a three or four from experts. A score is considered to have good validity 80% of the time. The planned questionnaire's CVI was determined. The response rate to the survey used in the study was 86%.

Reliability analysis 

An analysis of the reliability of internal consistency revealed a Cronbach's alpha value of 0.79.

Statistical analysis

Data analysis was implemented using RStudio (R version 4.1.1). Frequencies and percentages were used to express categorical data. A multiple-response analysis was used to analyze variables with multiple selections. Statistical differences between categorical variables were assessed using Pearson's Chi-squared test or Fisher's exact test whenever applicable. Statistical significance was considered at p<0.05.

Ethical approval

Ethical approval was obtained from the Medical Ethics Committee of Umm Al-Qura University, Saudi Arabia (HAPO-02-K-012-2022-11-1294).

## Results

Demographic and professional characteristics

**Figure 1:**
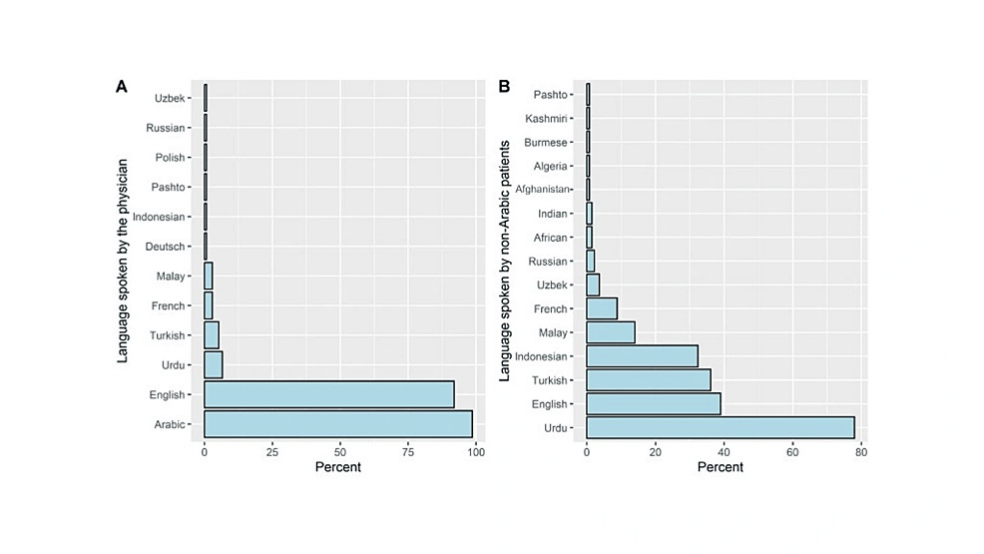
Language spoken by physicians (a) and language spoken by non-Arabic patients (b)

This study included 136 participants, the majority were males (70.6%; n=96) and residents (77.2%; n=105). Half of ED physicians had a professional experience of one to three years in the ED (Table 2). The majority of ED physicians were speaking Arabic or English as the main language (98.5% and 91.9%, respectively), whereas other spoken languages included Urdu (6.6%) and Turkish (5.1%, Figure 1A). The most common languages spoken by patients at ED were Urdu (77.9%), English (39.0%), Turkish (36.0%), and Indonesian (32.4%, Figure 1B).

**Table 2:** Demographic and professional characteristics *N.B. Resident service: physician who graduated from medical school but did not complete a post-graduate training program, working as a general physician; resident board: physician who graduated from medical school and completed a post-graduate training program. Specialist: a board-certified physician who completed their residency training. ED, emergency department

Parameter	Category	N (%)
Gender	Male	96 (70.6%)
	Female	40 (29.4%)
Position*	Resident service	105 (77.2%)
	Resident board	9 (6.6%)
	Specialist	19 (14.0%)
	Consultant	3 (2.2%)
Duration of practice in ED in Makkah, KSA (year)	<1	29 (21.3%)
	1 to 3	68 (50.0%)
	4 to 8	27 (19.9%)
	>8	12 (8.8%)

Characteristics of language barriers and the associated factors

Of note, 89.7% (n=122) of ED physicians under study stated that they always or sometimes experienced language barriers. Only 10.3% (n=14) of ED physicians had rarely or never experienced language barriers. Language barriers were significantly higher among specialists (100.0 vs 0.0%) and resident service (93.3% vs 6.7%, p<0.001) (Table 3).

**Table 3:** Factors associated with experiencing language barriers at the ED ED, emergency department

Parameter	Category	Experiencing language barriers		p-value
		Never or rarely, N=14	Sometimes or always, N=122	
Gender	Male	11 (11.5%)	85 (88.5%)	0.757
	Female	3 (7.5%)	37 (92.5%)	
Position	Resident service	7 (6.7%)	98 (93.3%)	<0.001
	Resident board	5 (55.6%)	4 (44.4%)	
	Specialist	0 (0.0%)	19 (100.0%)	
	Consultant	2 (66.7%)	1 (33.3%)	
Duration of practice in ED (year)	<1	2 (6.9%)	27 (93.1%)	0.180
	1 to 3	5 (7.4%)	63 (92.6%)	
	4 to 8	6 (22.2%)	21 (77.8%)	
	>8	1 (8.3%)	11 (91.7%)	

Information loss and poor outcomes due to poor communication

As for the ED physicians' opinion, 18.4% (n=25) reported that information loss always occurs, while 72.1% (n=98) and 8.1% (n=8.1) stated that it sometimes or rarely occurs. Only 1.5% (n=2) of them stated that information had never been lost upon communication with non-Arabic and non-English patients. More than half of the ED physicians (54.4%) stated that their patients had poor outcomes because of poor communication. Among emergency physicians who reported that the patient ever had poor outcomes because of poor communication (n=74), the most frequently reported poor outcomes included unimproved\worsening symptoms leading to recurrent visits to ED (60.8%; n=45) and patient dissatisfaction (45.9%; n=34, Table 4).

**Table 4:** Characteristics of information loss and poor outcomes due to poor communication *More than one answer was allowed (N.B.)

Parameter	Category	N (%)
Which of the following factors were associated with better outcomes in patient care?*	Professional interpreter	97 (71.3%)
	Body language	34 (25.0%)
	Asking for help from someone who speaks the same language	73 (53.7%)
	Translation application	21 (15.4%)
Which of the following factors were associated with poor outcomes in patient care?*	Using Google translator instead of an advanced translation application	43 (31.6%)
	Body language	70 (51.5%)
	Professional interpreter is not available	69 (50.7%)
	I did not face any poor outcomes	19 (14.0%)
	Asking for help from someone who speaks the same language	33 (24.3%)
	No one available that speaks the same language	1 (0.7%)
Suggestion for improving patient communication*	Providing labels of the common medical terminology in different languages	81 (59.6%)
	Providing foreign language communication courses	66 (48.5%)
	Using telephone interpreter	65 (47.8%)
	Translators who come with patients during hajj	1 (0.7%)
	Provision of translation device	1 (0.7%)
	Providing translators on-call	1 (0.7%)
	Professional translator	1 (0.7%)
	Professional interpreter	1 (0.7%)

The occurrence of poor outcomes due to poor communication did not differ significantly based on physicians’ gender, experience, or reporting language barriers and information loss when communicating medical information to non-Arabic and non-English patients. However, poor outcomes due to poor communication were significantly less frequent among consultants (0.0%) compared to residents (60.0%), resident board members (33.3%), and specialists (42.1%, p=0.048, Table 5).

**Table 5:** Factors associated with having poor outcomes due to poor communication ED, emergency department

Parameter	Category	Poor outcomes due to poor communication		p-value
		No, N=62	Yes, N=74	
Gender	Male	43 (44.8%)	53 (55.2%)	0.773
	Female	19 (47.5%)	21 (52.5%)	
Position	Resident service	42 (40.0%)	63 (60.0%)	0.048
	Resident board	6 (66.7%)	3 (33.3%)	
	Specialist	11 (57.9%)	8 (42.1%)	
	Consultant	3 (100.0%)	0 (0.0%)	
Duration of practice in ED (year)	<1	13 (44.8%)	16 (55.2%)	0.972
	1 to 3	30 (44.1%)	38 (55.9%)	
	4 to 8	13 (48.1%)	14 (51.9%)	
	>8	6 (50.0%)	6 (50.0%)	
Information loss when explaining medical information to non-Arabic and non-English patients	Never/rarely	8 (61.5%)	5 (38.5%)	0.225
	Always/sometimes	54 (43.9%)	69 (56.1%)	
Experiencing language barriers	Never/rarely	9 (64.3%)	5 (35.7%)	0.138
	Always/sometimes	53 (43.4%)	69 (56.6%)	

Method of communication and their influence on patients’ outcomes

**Figure 2:**
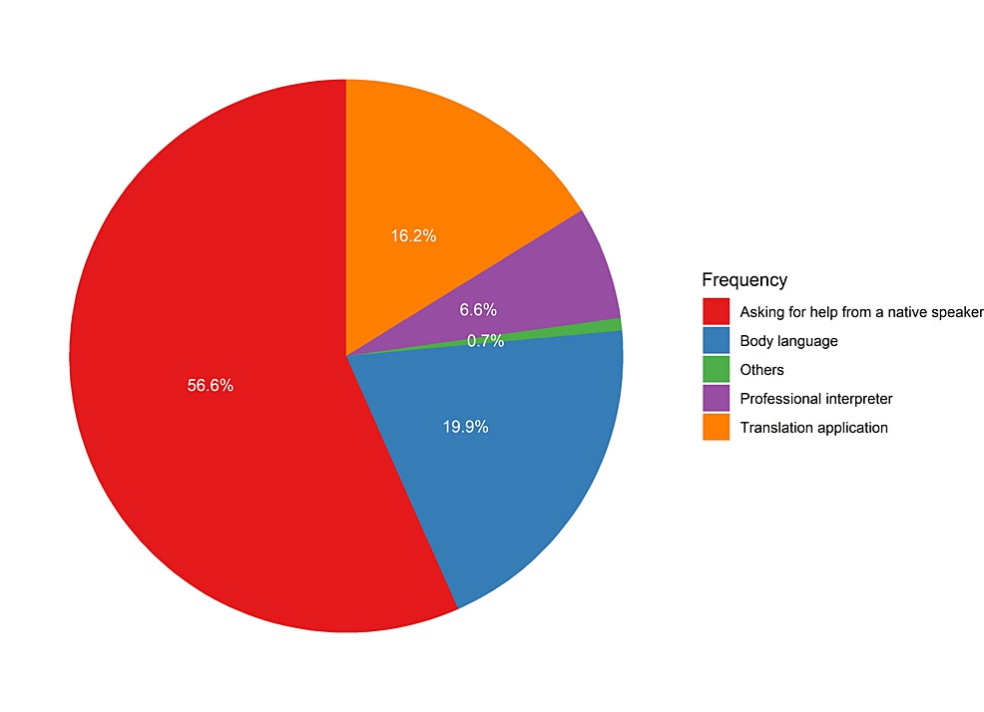
Method of communication with non-Arabic and non-English patients in ED ED, emergency department

The most used solution of communication with non-Arabic and non-English speaking patients was asking for help from a native speaker (56.6%, Figure 2). ED physicians choose such a method because it is easy to coordinate (64.9%), has a better quality (46.8%), and is low cost (20.8%). Although a professional interpreter was used by only 6.6% of ED physicians for communication (Figure 2), the majority of respondents in the current study (88.2%) stated that the existence of a professional interpreter is very important in the hospital.

When the participating ED physicians were asked about factors that could enhance the communication level between the physician and the patient, the most common methods to address language barriers were the existence of a professional interpreter (71.3%; n=97) and asking for help from a native speaker (53.7%; n=73). On the other hand, the most frequently reported factors that would be associated with poor outcomes were the use of body language (51.5%; n=70) and the lack of a professional interpreter (50.7%; n=69). ED physicians’ suggestions to improve communication with patients included providing labels of the common scientific terminologies in different languages (59.6%; n=81) and providing courses to communicate in foreign languages (48.5%; n=55) (Table 6).

**Table 6:** Factors associated with patients’ outcomes and physicians’ suggestions to improve communication with patients *More than one answer was allowed (N.B.)

Parameter	Category	N (%)
Which of the following factors were associated with better outcomes in patient care?*	Professional interpreter	97 (71.3%)
	Body language	34 (25.0%)
	Asking for help from someone who speaks the same language	73 (53.7%)
	Translation application	21 (15.4%)
Which of the following factors were associated with poor outcomes in patient care?*	Using Google translator instead of advanced translation application	43 (31.6%)
	Body language	70 (51.5%)
	Professional interpreter is not available	69 (50.7%)
	I did not face any poor outcomes	19 (14.0%)
	Asking for help from someone who speaks the same language	33 (24.3%)
	No one available that speaks the same language	1 (0.7%)
Suggestion for improving patient communication*	Providing labels of the common medical terminology in different language	81 (59.6%)
	Providing foreign languages communication courses	66 (48.5%)
	Using telephone interpreter	65 (47.8%)
	Translators who come with patients during hajj	1 (0.7%)
	Provision of translation device	1 (0.7%)
	Providing translators on-call	1 (0.7%)
	Professional translator	1 (0.7%)
	Professional interpreter	1 (0.7%)

## Discussion

Communication barriers in the ED are challenging to have effective physician-patient communication, considering the noise, lack of privacy, frequent interruptions, limited time, staff and resource shortages, unpredictability, overcrowding, and increased patient admissions. In addition to the complicated situations encountered by emergency physicians, including death, rape, acute illness, and accidents, the biggest obstacle to providing adequate, appropriate, effective, and timely care to patients who do not speak the local language is language barrier [1,4].

Based on the results of our study, the exposure to the language barrier in the ED was high as 89.7% of physicians under study stated that they always or sometimes experienced language barriers. This high level of language barriers was expected as Makkah is considered a multicultural city because of Hajj and Umrah. Prior studies showed that language limitations made it challenging to interact with patients and their families [14]. The prevalence of language barrier in the present study was higher than that observed in a study conducted in Norway, where medical providers had difficulty understanding between 36% and 43% of the patients who did not speak the local language, necessitating the need for interpreters [8].

In the current study, 18.4% of the ED physicians reported that information loss always occurs, while 72.1% and 8.1% stated that it sometimes or rarely occurs. This was explained in previous studies by the language difficulties, cultural variations, and overcrowding in ED [2,15]. In an Indian ED survey, 64% of respondents thought that information was lost or altered when English medical knowledge was delivered in a different language [2]. In a separate study, almost half of the physicians surveyed in a trilingual emergency room in Hong Kong expressed concern that repeated translation could lead to the omission or alteration of medical information [15].

Our study showed a high dependence on asking for help from a native speaker in communication with non-Arabic and non-English patients. This is reasonable, as such methods are easy to coordinate and have low costs. It might not be considered helpful all the time, as a native speaker can deliver the information without emotional involvement, which may lead to translational errors contrary to friends and family since interpretation can be hard on them due to embarrassment and sociocultural taboos depending on the presenting issue [1]. A matter that causes an ethical dilemma as it involves disclosing medical information of the patient to another person. The research suggests that it is beneficial to select a nurse or other healthcare provider who is fluent in the patient's language and has had their language abilities professionally tested by a language assessment expert [16]. In addition to appearing inadequate for significant medical contacts, the use of Google Translate to overcome language barriers appeared to be of limited assistance in emergencies or in dealing with emotional distress. A recent study shows that it is not a replacement for bilingual care or expert interpreter services, while being easily accessible and possibly effective in some circumstances [17].

In the present study, according to the participant ED physicians the most commonly mentioned solutions to overcome the language barriers for better patients’ outcomes were the existence of a professional interpreter and asking for help from a native speaker. In addition, ED physicians suggested providing labels for the common scientific terminologies in different languages and providing courses to communicate in foreign languages to improve communication with patients. In contrast, the Swiss study showed that regular use of brochures and visual aids to help with a language barrier is quite rare [18]. According to a study conducted in Canadian provinces, language barriers contribute to poorer quality of care and patient safety [17]. In our study we found out that one of the most important returns of language barriers, according to ED physicians' indications, is information loss, which could occur sometimes (72.1%; n=98); therefore, more than half of the ED physicians (54.4%; n=74) stated that their patients had poor outcomes because of poor communication. This poor outcome was highlighted in a previous study conducted in Addis Ababa, where language barriers were reported to cause avoidable medical errors, poor treatment adherence, low health-seeking behavior, additional treatment costs, longer hospital stays, weak therapeutic relationships, social desirability bias, a lack of confidence, and dissatisfaction with the quality of care [19].

As for the presence of professional interpreters to solve the language barriers, a previous systematic review revealed that the use of professional interpreters was correlated with better healthcare quality for patients with limited English ability [20]. It has been shown to reduce health disparities by reducing errors and improving access and satisfaction [21].

There are different ways to improve language barriers. In this study, more than half of ED physicians suggested providing labels of common medical terminology in different language methods to decrease language barrier effects. Moreover, less than half suggested providing foreign language communication courses and using a telephone interpreter.

The second factor that played a role in miscommunication after language was an increased number of patients [22]. The process of efficient communication between the ED physician and the patient is weakened as the number of patients increases due to the decreased quality of communication between the two parties [22]. Additionally, the number of patients admitted to the emergency room varies and is not predictable. This may affect the appropriate time for each patient and increase anxiety, stress, and inefficient work.

Developed countries, including the Kingdom of Saudi Arabia, aim to develop and improve health services for individuals. Despite the small sample size in this study, the results highlighted one of the problems facing healthcare providers, which may obstruct the achievement of high-quality health services. Furthermore, it offers a chance for advancement in the health field in combination with technical innovation.

Our study has several strengths as it was conducted in Makkah hospitals, which witness pilgrims from all over the world, especially during the Umrah season in Ramadan and during the Hajj. We also included ED physicians with different experiences and ranks (residents, specialists, and consultants). Our study is the first to address the existence of language and communication barriers between ED physicians as well as give an insight into possible solutions to this problem in ED in Makkah. Using a professional interpreter, providing labels of the common scientific terminologies in different languages, and providing courses to communicate in foreign languages may improve the quality of healthcare and the level of satisfaction among both medical providers and patients.

Limitations

Our study limitations included that we only conducted our research in MoH hospitals in Makkah in the ED. Also, there were a few studies that discussed language barrier problems in Saudi Arabia.

## Conclusions

The language barrier in the ED of the MoH Hospitals in Makkah was high, which may affect patient care. ED physicians use multiple strategies to overcome this problem, including communication with patients through native speakers. Other strategies were recommended to enhance ED physician-patient communication, like providing labels for the most commonly used medical terms in different languages. It is recommended that more studies be conducted to better understand and detect the difficulties that patients face due to the presence of language and communication barriers with healthcare providers in other regions of Saudi Arabia as well as the extent of their satisfaction with the quality of healthcare in the presence of these barriers. Additionally, since the number of pilgrims in religious sites such as Makkah and Madinah is continuously increasing, a study regarding language barriers among healthcare providers at the ED in both sites is necessary during that period or shortly after to address that issue. Furthermore, future studies that address the impact of poor communication on patients' outcomes and how to deal with situations of language barriers are recommended.
